# Nonverbal Semantics Test (NVST)—A Novel Diagnostic Tool to Assess Semantic Processing Deficits: Application to Persons with Aphasia after Cerebrovascular Accident

**DOI:** 10.3390/brainsci11030359

**Published:** 2021-03-11

**Authors:** Katharina Hogrefe, Georg Goldenberg, Ralf Glindemann, Madleen Klonowski, Wolfram Ziegler

**Affiliations:** 1 Clinical Neuropsychology Research Group, Institute of Phonetics and Speech Processing, Ludwig-Maximilians-Universität München, 80799 Munich, Germany; ralf.glindemann50@gmail.com (R.G.); wolfram.ziegler@ekn-muenchen.de (W.Z.); 2 Neurologische Klinik und Poliklinik, Klinikum Rechts der Isar, Technische Universität München, 81675 Munich, Germany; georg.goldenberg@tum.de; 3 Sprach- und Schlucktherapie, Schön Klinik München Schwabing, 80804 Munich, Germany; MKlonowski@schoen-klinik.de

**Keywords:** assessment tool, semantic processing, aphasia, semantic sorting, pantomime, drawing

## Abstract

Assessment of semantic processing capacities often relies on verbal tasks which are, however, sensitive to impairments at several language processing levels. Especially for persons with aphasia there is a strong need for a tool that measures semantic processing skills independent of verbal abilities. Furthermore, in order to assess a patient’s potential for using alternative means of communication in cases of severe aphasia, semantic processing should be assessed in different nonverbal conditions. The Nonverbal Semantics Test (NVST) is a tool that captures semantic processing capacities through three tasks—Semantic Sorting, Drawing, and Pantomime. The main aim of the current study was to investigate the relationship between the NVST and measures of standard neurolinguistic assessment. Fifty-one persons with aphasia caused by left hemisphere brain damage were administered the NVST as well as the Aachen Aphasia Test (AAT). A principal component analysis (PCA) was conducted across all AAT and NVST subtests. The analysis resulted in a two-factor model that captured 69% of the variance of the original data, with all linguistic tasks loading high on one factor and the NVST subtests loading high on the other. These findings suggest that nonverbal tasks assessing semantic processing capacities should be administered alongside standard neurolinguistic aphasia tests.

## 1. Introduction

Semantic cognition allows us to understand and interpret words and objects that we encounter in everyday life. It is essential for human communication, e.g., for initiating verbal utterances as well as nonverbal expressions like gestures or drawings. In persons with brain damage, it can be compromised.

The most common tasks to assess semantic processing in patients with neurologic conditions rely on verbal processing capacities, as is the case in confrontation naming and category fluency for production, or spoken word to picture matching for comprehension. These task types are part of many diagnostic tools, for example the CERAD battery, Consortium to Establish a Registry for Alzheimer’s Disease [1] and the MoCA Test, Montreal Cognitive Assessment [2] for dementia, or the Aachen Aphasia Test, AAT [3] and the Western Aphasia Battery—Revised [4] for aphasia after stroke. Persons with damage to the left hemisphere and aphasia often show deficits in these tasks. However, these deficits do not allow full conclusions to be drawn on the functional locus of impairment. Disturbed performance in verbal comprehension tasks may be due to word-form-level deficits and to impaired semantic cognition [5,6]. In production, the attempt to name a picture may result in null reactions or different types of incorrect realizations like phonemic paraphasias, semantic paraphasias, or neologisms. To determine the functional locus of impairment a number of different tasks and analyses may be applied, e.g., to disentangle the origin of semantic paraphasias or null reactions. Since these diagnostic strategies are not always successful, the use of non-verbal tasks could help to determine if impairments originate at the level of semantic cognition.

The most common approach relies on picture-based tasks that may be categorized as semantic sorting tasks, such as the Pyramids and Palm Tree Test, PPT [7], and the—more sensitive—Camel and Cactus Test, CCT [8,9]. Both require the recognition of associative relationships. Some of their items are culture-specific and, despite being non-verbal, cannot easily be transferred to other language communities. In the German-speaking area, a widely-used test based on an odd-one-out paradigm is the Bogenhausener Semantik-Untersuchung [10].

In addition to these tasks, which are largely receptive since they require only pointing, semantic processing capacities can also be assessed in expressive modalities like drawing or the execution of meaningful gestures [11]. Traditionally, these tasks are used in neuropsychology to examine other cognitive abilities. Drawing is mainly used for the assessment of visuo-constructional abilities or neglect [12] but has already also been used to determine the integrity of semantic concepts in patients with semantic dementia [13] as well as in persons with Alzheimer’s-type dementia [14]. Only recently, a brief drawing task consisting of only four items has been developed with the aim of differentiating between different dementia subtypes [15]. In persons with aphasia (PWA) after stroke, drawing tests have been used to assess object representations [16,17]. The production of gestures and in particular the production of Pantomime of object use is predominantly used in limb apraxia assessment, e.g., [18,19,20]. However, one may take the perspective that impairments of Pantomime of object use that motivate a diagnosis of apraxia actually reflect impaired semantic processing, because pantomimes require manual actions to convey information about the shape of objects and their use [21,22]. In this sense, Pantomime of object use discloses impairments of semantic processing capacities [23].

Also, in studies concerned with the clinical assessment and treatment of aphasia, drawing and gesture have been present for a long time, e.g., [24]. Only recently new assessments of functional communication were developed in which these two modalities are scored on an equal footing with spoken language, i.e., the Scenario Test [25,26,27], and the German KOPS, Kommunikativ-pragmatisches Screening [28]. Moreover, drawing and gesture play a role in speech and language therapy. For both, facilitatory effects on word retrieval have been explored in treatment studies [29,30,31,32], for gesture observation [33,34]. Furthermore, drawing and gesture have long played an important role as compensatory expressive means in aphasia therapy [35,36,37,38]. Hence, apart from giving insight into semantic processing abilities, the assessment of drawing and gesture abilities allows for the determination of the potential of these communication channels for PWA.

Assessing semantic processing capacities with different nonverbal tasks is in line with theories that assume modality-specific semantic processing. A recent approach—the hub-and-spoke hypothesis—that incorporates many aspects of previous theoretical accounts assumes an interaction of modality-specific cortical regions with an amodal central representational hub whenever semantic processing occurs [39,40,41,42].

We recently developed the Nonverbal Semantics Test, NVST [43], that comprises the three above-mentioned task types—Semantic Sorting, Drawing, and Pantomime. The Nonverbal Semantics Test is a standardized assessment tool for the clinical assessment of semantic processing disorders in persons with neurological disorders (cerebrovascular accident (CVA) and neurodegenerative disease). Results allow comparisons between the subtests and indicate if particular nonverbal resources are intact and can be used for successful functional communication. The results may form the basis for tailored therapy planning and can be used for monitoring therapy outcomes. In persons with dementia, the test may support differential diagnosis of dementia subtypes. The Appendix A contains a detailed description of the test.

One aim of the present study was to investigate how persons with moderate to severe aphasia in the acute to chronic phase of the language disorder perform in the NVST subtests and how the subtests are related to each other. Assuming that semantic information is processed in a modality-specific manner, we hypothesized that the three NVST subtests would be impaired to different degrees, resulting in different patterns of performance in the NVST. The second aim was to determine the relationship of the NVST subtests with standard measures of neurolinguistic processing. To test the hypothesis that the three subtests of the NVST are indicators of a trait that is separate from aphasia, a principal component analysis was used to identify the main dimensions of variance when the NVST variables are pooled together with neurolinguistic measures of aphasia.

## 2. Materials and Methods

Participants: Participants were recruited in cooperation with several local clinical institutions (see Acknowledgements). Fifty-one persons with left hemisphere damage participated in the present study (26 female; mean age 61 years, range 29–82, standard deviation (sd) = 12.4). Forty-nine participants had suffered a unilateral cerebrovascular accident (ischemic infarction: n = 40 or hemorrhagic infarction: n = 9), whereas two had traumatic brain damage resulting in focal lesions. All participants were at least two weeks post-onset (mean 18, range 2 weeks—198 months, sd = 33.5) and had no additional significant neurological conditions and no auditory perception deficits according to clinical records. All patients were rated between zero and three on the verbal communication scale of the spontaneous speech evaluation of the Aachen Aphasia Test [3] (AAT six-point-scale for verbal communication (paraphrased and shortened from AAT manual): 0: no comprehensible utterance production and manifest impairments in comprehension; 1: PWA communicates through incomplete, mostly incomprehensible utterances; the listener has to guess or ask for more information; 2: talking about familiar topics is only possible with help of the communication partner, but the PWA is frequently unable to convey the message; 3: talking about familiar topics is possible with little support of the communication partner and communication is markedly impaired; 4: fluency of language production is reduced and/or some verbal difficulties are present; 5: no or minimal impairment in verbal communication). For a description of the AAT compare also [44]. This criterion was applied as the NVST is considered particularly suitable for persons with moderate to severe aphasia.

Clinical assessment of aphasia: The Aachen Aphasia Test was administered to all 51 participants. It consists of an evaluation of spontaneous speech and has five subtests—Token Test, naming, comprehension, repetition, and written language. T-normalized scores were determined. Aphasia type was classified according to the AAT protocol and in two cases according to clinical impression. Six participants presented with anomic aphasia (12%), ten with Broca’s aphasia (19.5%), ten with Wernicke’s aphasia (19.5%), and 23 with global aphasia (45%). One participant was diagnosed with mixed transcortical aphasia (2%) and one with transcortical sensory aphasia (2%).

Assessment of nonverbal semantic processing: The NVST was administered to all participants. It consists of the subtests Semantic Sorting (requiring participants to recognize semantic relationships in black and white line drawings), Drawing (requiring participants to depict salient visual features of an object), and Pantomime (requiring participants to demonstrate the use of an object). A detailed description of the test is given in the Appendix A. Standardized scores were determined using non-parametric methods, as specified in the Appendix A.

Statistical analysis: Pearson correlations were calculated between the subtests of the NVST as well as between the subtests of the NVST and standard neurolinguistic tasks (AAT). Furthermore, multiple linear regression analyses were performed for each NVST variable separately, with the AAT subtests as regressor variables. Finally, the NVST standardized scores and the AAT T-normalized scores were subjected to a principal component analysis (PCA) with varimax rotation. The standard Kaiser criterion (extract factors with eigenvalue >1.0) was used to determine the factors that captured meaningful variance in the data. All statistical analyses were performed using R [45].

## 3. Results

### 3.1. Performance in the NVST Subtests

#### 3.1.1. Degrees of Impairment in the NVST Subtests

The scores obtained in each of the three NVST subtests are listed in Table 1 (left-most column, shaded). A linear model was calculated using the function ‘lm’ of the R-package ‘lme4’ to compare the standardized NVST scores across the three subtests, with the question being whether the three NVST modalities of semantic processing are equally impaired in patients with aphasia. Testing the model fit using the ANOVA function of R revealed a significant F-value of 6.48 (*p* < 0.01). Beta coefficients of Pantomime vs. Semantic Sorting and Drawing were 0.85 and 0.84, respectively, (*p* < 0.01 in both cases), while Semantic Sorting and Drawing were not different (β = 0.01, *p* > 0.05). Table 1 also displays the classification of PWA according to four severity levels based on coarser-grained NVST distance metrics (see Appendix A). Whereas in Semantic Sorting and Drawing most of the participants showed no or only mild impairment, Pantomime revealed a high number of moderately impaired participants.

#### 3.1.2. Relationships between NVST Subtests

**Figure 1:**
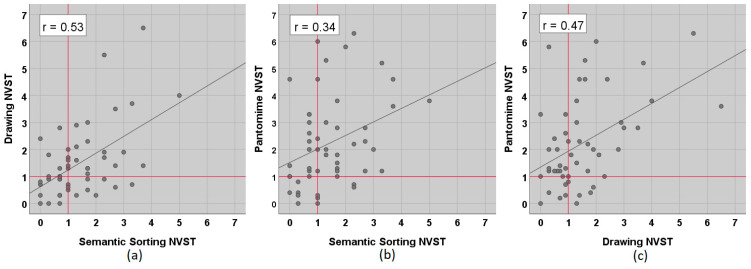
Relationship of the three subtests of the Nonverbal Semantics Test (NVST). (**a**) Drawing vs. Semantic Sorting, (**b**) Pantomime vs. Semantic Sorting, and (**c**) Pantomime vs. Drawing. Data points represent normative scores. High scores indicate a more impaired performance. Red lines mark the cut-off (1).

At the individual level, Figure 1 shows that the examined PWA were not impaired consistently across tasks. Although the three NVST subtests were moderately correlated with each other (Pearson, all *p* < 0.05, compare Figure 1), there were double dissociations in all three comparisons, with several patients obtaining scores within the normal range (i.e., <1) in one task and substantially increased scores in the other. As documented in Table 1, more participants were impaired in the Pantomime task as opposed to Semantic Sorting or Drawing, but a sparing of the Pantomime abilities did not necessarily entail a sparing of Semantic Sorting or Drawing capacities (middle and right panels of Figure 1).

### 3.2. Relationship of NVST Subtests with Standard Neurolinguistic Measures (AAT)

#### 3.2.1. Correlations between the Subtests of the NVST and the Subtests of the AAT

Correlations of the NVST subtests with the AAT subtests are tabulated in Table 2. For Semantic Sorting, significant correlations were obtained with Comprehension, for Drawing with Written Language and Comprehension, and for Pantomime with all AAT subtests. However, all correlations were at best weak to moderate.

#### 3.2.2. Linear Regression Models

In order to determine whether the NVST scores can be predicted from the patients’ performance in the AAT testing, a linear regression model was calculated for each of the three NVST subtests. Computation of the variance inflation factors for the five AAT variables using the ‘vif’ function in R [46] revealed that all vif coefficients were clearly below 4.0, indicating that multicollinearity was not a problem in these analyses [47] (page 102). Model fit was tested using the ANOVA function in R. A significant F-value was only obtained for the model of the Pantomime scores (F(5, 45) = 4.58, *p* < 0.01), with a significant β-coefficient for only the Comprehension subtest of the AAT (β = −0.07; note that poorer performance yielded higher NVST standardized scores and lower AAT T-values). The linear models for the Semantic Sorting and the Drawing tasks failed to fit the data (F(5, 45) = 1.07 and 2.19, respectively; *p* > 0.05), and, consequently, none of the AAT scores obtained a significant β-coefficient.

**Figure 2:**
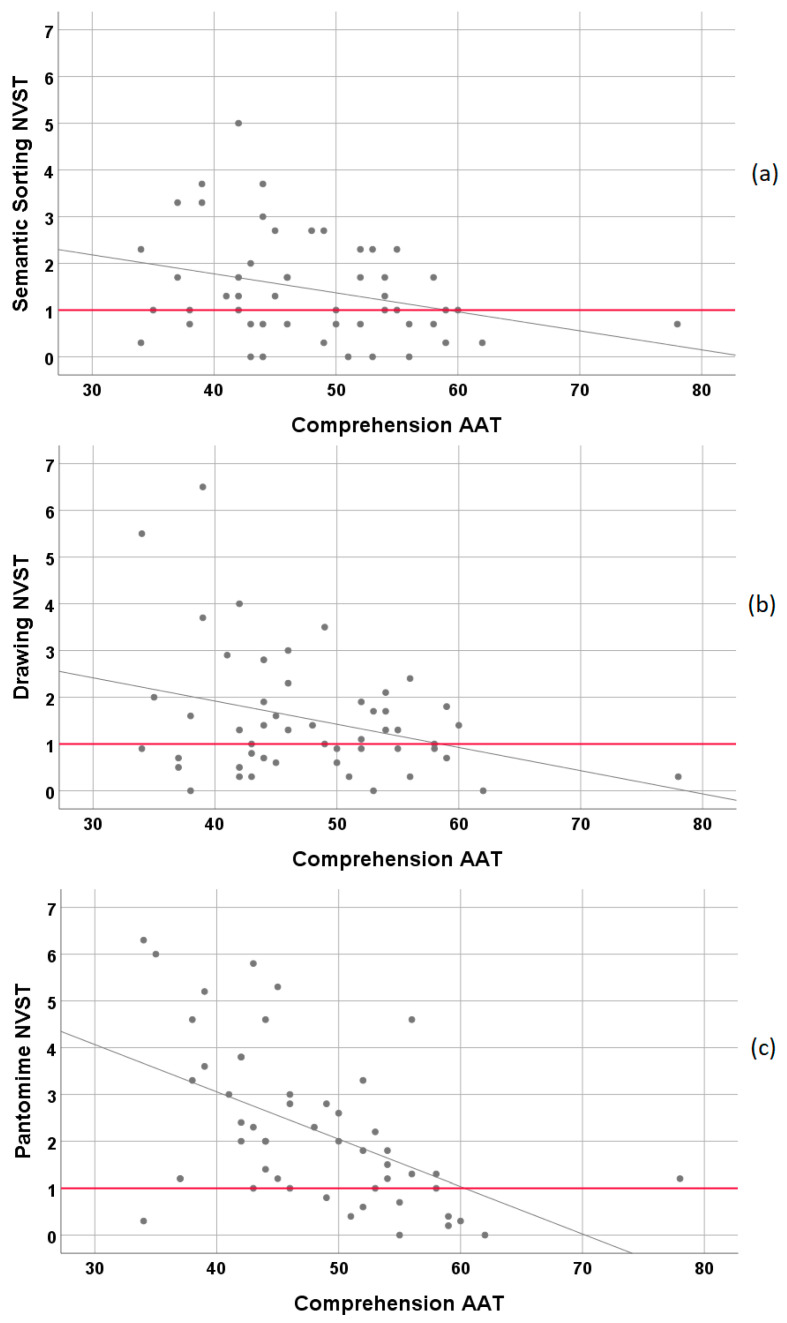
Relationship of NVST subtests with subtest Comprehension of the AAT. (**a**) Semantic Sorting vs. Comprehension, (**b**) Drawing vs. Comprehension, and (**c**) Pantomime vs. Comprehension. For correlation coefficients see Table 2.

Figure 2 illustrates the relationship of the AAT-subtest Comprehension with the three subtests of the NVST. It demonstrates that semantic processing, as measured by the NVST subtests, may be unimpaired in persons with even severe language comprehension impairment according to the AAT scores.

#### 3.2.3. Principal Component Analysis

**Figure 3:**
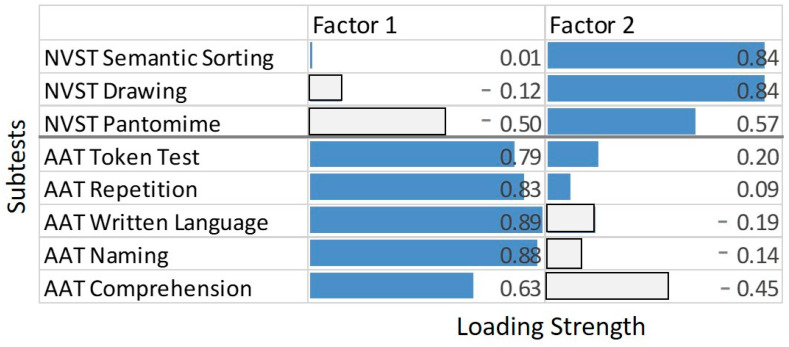
Principal component analysis (PCA) results. The two panels display the factor loadings (absolute values) of each task on the two factors. Dark shaded bars represent indicators with absolute loadings >0.50. Factor 1 explained 44% and factor 2 25% of the variance in the data.

In order to elucidate how the NVST variables combined with the conventional aphasia scores of the AAT to a lower-dimensional description of the variance, a factor analysis was conducted. It was hypothesized that the AAT indicators of neurolinguistic impairment and the NVST indicators of semantic impairment were largely independent. Since the sample size was too small to conduct a confirmatory factor analysis with two latent variables loading onto the neurolinguistic and the semantic indicators, respectively, an exploratory factor analysis was chosen. More specifically, a principal component analysis (PCA) with factor extractions based on the Eigenvalue criterion was computed, including the five AAT subtests and the three NVST subtests as independent variables (i.e., with a cases to variable ratio of >6). Testing the sampling adequacy of the data set using the ‘KMO’ function of R revealed a highly satisfactory overall MSA of 0.78, with all individual MSA coefficients >0.6. The PCA, conducted using the ‘principal’ function of the ‘psych’ package in R [48], resulted in a two-factor solution explaining 69% of the variance in the original data. After a varimax rotation, the factor loadings indicated a clear separation between the five standard neurolinguistic measures of aphasia (AAT), on the one hand, and the three NVST variables, on the other. Factor 1 had high positive loadings on the AAT variables and much lower loadings, in absolute terms, on the three NVST variables, while the reverse was true for factor 2 (Figure 3). Note, however, the areas of strain on the NVST Pantomime and the AAT Comprehension variables in the PCA model of Figure 3, both of which received loadings that were less compatible with the strict separation between AAT- vs. NVST-related factors.

## 4. Discussion

### 4.1. How Do Persons with Moderate to Severe Aphasia Perform in the NVST and How Are the Subtests Related to Each Other?

All 51 PWA were able to complete the NVST subtests. Hence, the test can easily be administered even in persons with severe aphasia and in the acute phase of their disorder. The three subtests of the NVST were moderately correlated with each other. However, individual PWA were not equally impaired across the NVST subtests—double dissociations occurred in all three comparisons, with several PWA showing normal performance in one task and a markedly impaired performance in the other. This finding may support the notion of modality-specific processing of semantic information and underlines that the three NVST subtests capture different aspects of semantic processing. It also shows that it is not recommended to calculate a composite score across the three subtests, as the identification of different patterns of performance provides important information that may guide therapy planning.

At the group level, impairments in the three different subtests varied in severity: The performance in the subtest Pantomime was markedly more impaired as opposed to Semantic Sorting or Drawing. The finding that our sample was more impaired in Pantomime as opposed to Drawing replicates results of a study that applied previous versions of the NVST subtests Drawing and Pantomime to 40 PWA [17].

### 4.2. How Do the NVST Subtests Relate to Standard Neurolinguistic Measures?

All NVST subtests were correlated on a significant level with the AAT subtest Comprehension. Moreover, in contrast to Semantic Sorting and Drawing, Pantomime was related to all standard measures of neurolinguistic processing as measured with the AAT. This observation seems to be a robust finding since it has been reported several times in studies that used the previous version of the NVST subtest Pantomime e.g., [17,49]. Recently, it has been proposed that the production of Pantomime relies on two core neural networks combining motor-cognitive and communicative aspects of performance. The observation of a moderate relationship between neurolinguistic measures and Pantomime could be explained by this account. For an extensive discussion of this proposal compare Finkel, et al. [50]. Another factor that might contribute to the association between neurolinguistic capacities and pantomime is the prevalence of its combination with verbal expression. In line with the “gesture first” theories of the origins of language that claim that pantomime preceded speech as an initial form of referential communication [51,52,53], it might be speculated that their common use in interpersonal communication has led to close functional and anatomical proximity, and hence similarity of the effects of localized brain damage.

The observed correlative pattern was substantiated by the PCA presented here, which revealed a clear separation between a factor representing the standard neurolinguistic capacities measured by the AAT variables and a second factor representing the nonverbal semantic capacities measured by the three NVST variables. Notably, however, the factor loadings on the NVST subtest Pantomime and the AAT subtest Comprehension were less compatible with this overall picture of a strict separation between a “neurolinguistic” and a “nonverbal semantic” trait as it also became obvious in the regression analysis, where Comprehension was the only predictor of Pantomime.

The results of the PCA suggest that the NVST measures different aspects than standard neurolinguistic measures like the AAT. The application to PWA (especially to persons with severe aphasia) can support a comprehensive assessment and may help to determine if impairments originate at the level of semantic cognition.

In a recent study [54], 99 persons with mild to moderate chronic aphasias were assessed on 17 different measures that covered speech perception and production as well as verbal (word-level) and nonverbal cognition. A PCA revealed a model with four factors explaining 76% of the variance in the data—two “speech” factors (related to productive and receptive phonological processes), a “semantic errors” factor loading exclusively on a variable that counted semantic errors in confrontation naming, and a further factor of “semantic recognition”, with high loadings on tasks that are generally understood to explicitly require semantic processing capacities, e.g., verbal tasks like word to picture matching, synonym judgments, or confrontation naming along with two nonverbal tasks, i.e., PPT and CCT. These data confirm that, at least in persons with less severe aphasic impairments, specifically designed verbal tasks (e.g., synonym judgments) or functionally focused analyses of error patterns (e.g., separate counts of semantic and phonological errors in picture naming) can reveal semantic processing deficits and generate data that go together with nonverbal semantic processing data. However, patients with severe language impairment, such as those included in the present study, may perform poorly on such measures for many reasons other than semantic impairment. This is precisely why nonverbal tests such as the NVST are a necessary tool for the clinical diagnosis of semantic abilities in PWA.

### 4.3. Why Is Pantomime more Impaired than Semantic Sorting and Drawing?

At the group level, the performance in the Pantomime task was markedly more impaired than the performance in the other two tasks. As compared to the mainly receptive Semantic Sorting task, Pantomime is a productive task that requires the retrieval of adequate features and their actual execution. As such the Pantomime task may per se be more error-prone than Semantic Sorting. However, it is also more impaired than the productive Drawing task. Whereas Drawing requires the depiction of aspects of the visual appearance of an object, the production of a Pantomime requires the depiction of visual aspects of the shape of an object in combination with aspects of motion. Hence, different types of content must be depicted with own body movements.

Furthermore, hemispheric lateralization may play a role for the performance patterns we observed in our sample. It is beyond the scope of this study to make detailed statements about neuroanatomical relationships with the performance in the NVST, but some more general aspects of hemispheric lateralization will be discussed, nonetheless.

The semantic system has often been described as depending predominately on a left hemispheric network, e.g., [55,56]. However, it was proposed that a less extensive semantic network exists in the right hemisphere, although the functional and anatomical differences between left and right brain semantic systems are still under debate [57]. There is broad agreement that the anterior temporal lobes (ATL) are important for the processing of conceptual knowledge, e.g., [41]. Again, the precise contribution of each side is debated, but there is consensus though that verbal semantic information is processed in the left ATL and some authors argue that the right ATL plays a role for the processing of non-verbal information—in particular for information concerning faces and objects, e.g., [11,58]. Compare also [41] for a detailed discussion. Concerning the NVST-tasks, different contributions of the right hemisphere are hypothesized, as described in the following paragraphs.

For Semantic Sorting there is evidence that the right hemispheric semantic system is involved in the processing of this task. Most relevant here is a study by Butler, et al. [59] that investigated the neural correlates of verbal and nonverbal semantic processing in a large sample of persons with neurodegenerative diseases using voxel-based morphometry. In this study, the PPT [7] was used in both the word and the picture version. The latter is similar to the NVST subtest Semantic Sorting in its requirement to make semantic decisions for line drawings. This study showed that regardless of the PPT version, semantic processing capacities were correlated with atrophy in both temporal lobes. Of note, for persons with semantic dementia, larger atrophy in the right ATL was associated with more impairment in the PPT picture version [59,60,61] suggesting differential roles of the two hemispheres for the processing of this task.

A contribution of the right hemisphere has also been described for Drawing. Whereas magnetic resonance imaging (fMRI) studies revealed activation of a large left hemispheric semantic network for simulated drawing [62], two other studies also showed considerable involvement of the right hemisphere for simulated drawing or drawing with a finger in the air [63,64,65]. Furthermore, persons with right hemisphere damage (RBD) were found to be more impaired in Drawing than PWA [17,66], and in PWA drawing capacities were shown to be independent of the type and severity of aphasia [16]. These findings suggest a contribution of the right hemisphere to the production of representational drawings.

Likewise, for gesture production, a role of the right hemisphere has been suggested. However, a study with patients with callosal disconnection [67] as well as studies with patients after CVA [68,69,70,71] showed that different gesture types originate from different loci in the brain. For the gesture type that is assessed in the NVST, namely Pantomime of object use, there is abundant evidence that it is a specific left hemispheric function [50,72,73]. For a comprehensive review on the nature and localisation of Pantomime compare Goldenberg [21].

To conclude, for the execution of the Semantic Sorting and Drawing tasks PWA may profit from bilateral networks, whereas their performance in the Pantomime task—that mainly relies on left hemisphere processes—is more compromised.

This assumption also fits with the results we obtained in a previous study with a smaller group of PWA and persons with dementia [74] in which we showed that the NVST subtests were sensitive across both groups, but with a significant interaction of group by subtest. This interaction resulted from a greater vulnerability of the Pantomime task in PWA as compared to dementia patients.

### 4.4. Implications for Treatment Planning

The identification of the different patterns of performance can support tailored treatment planning. Considering NVST results together with the performance on standard neurolinguistic measures can inform whether it is reasonable to include gesture and drawing in the therapy protocols. Especially for persons with severe aphasia and a highly reduced verbal output, gestures and drawing can be important resources for communication. For gesture, it has been shown that the performance in a Pantomime task predicts the comprehensibility of spontaneously produced meaningful gestures in persons with severe aphasia [75,76,77]. Hence, a relatively preserved performance in this subtest can indicate if a person will benefit from the use of gesture in communication and may therefore motivate therapists to initiate and reinforce the use of gestures [78]. The same holds for Drawing—the score in the NVST subtest Drawing reflects if this mode of expression has the potential to support the conveyance of information for persons with severe aphasia and if so, it should be included into the treatment protocol [79].

### 4.5. Limitations of the Present Study

It was beyond the scope of this study to investigate and address the question of a distinction between impaired semantic representations and impaired semantic access mechanisms that has been discussed extensively for PWA, e.g., [39,80,81]. For an overview of this discussion and a study that may disprove some of the accounts of the impaired access theories compare Chapman, et al. [82]. Additionally, and partly related to the aforementioned aspect, the impact of neuropsychological disorders that can accompany aphasia, like executive disfunctions or visuo-constructive disorders, have not been addressed. Whereas executive disfunctions have a more general impact on the performance in a range of different tasks, visuo-constructive disorders may have a particular influence on drawing tasks. Indeed, such an influence on the NVST Drawing task was found in persons with dementia [74].

## 5. Conclusions

The Nonverbal Semantics Test (NVST) is a standardized tool for the clinical assessment of nonverbal semantic abilities. It can be used without problems even for participants with severe language disorders. The NVST supports a detailed assessment of the underlying locus of deficit in PWA. Our data suggest that it measures abilities that are not captured by standard neurolinguistic parameters. Furthermore, the NVST allows for the description of performance patterns that indicate the potential of different non-verbal communication channels. The Nonverbal Semantics Test should be administered along with aphasia assessment to enable a tailored treatment planning.

## Figures and Tables

**Table 1:** Degree of impairment across tasks. First column (shaded), mean (sd) of normalized scores; columns 2 to 4, numbers of participants for each severity level (see Table A1 in the Appendix A).

	Mean (sd)	No Impairment	Mild Impairment	Moderate Impairment	Severe Impairment
Semantic Sorting	1.45(1.12)	18	19	13	1
Drawing	1.52(1.32)	21	18	9	3
Pantomime	2.24 (1.67)	10	15	18	8

**Table 2:** Results for the calculation of the correlation (Pearson) of the subtests of the NVST with standard neurolinguistic measures (AAT) for 51 persons with aphasia (PWA). Negative correlations reflect that high scores indicate severe impairment in the NVST, but mild impairment in the AAT.

	Semantic Sorting	Drawing	Pantomime
Token Test	*r* = −0.18, *p* = 0.21	*r* = −0.20, *p* = 0.15	***r* = −0.43, *p* < 0.01**
Repetition	*r* = −0.04, *p* = 0.81	*r* = −0.07, *p* = 0.68	***r* = −0.38, *p* < 0.01**
Written Language	*r* = −0.13, *p* = 0.37	***r* = −0.35, *p* < 0.05**	***r* = −0.46, *p* < 0.01**
Naming	*r* = −0.15, *p* = 0.29	*r* = −0.24, *p* = 0.09	***r* = −0.48, *p* < 0.01**
Comprehension	***r* = −0.31, *p* < 0.05**	***r* = −0.32, *p* < 0.05**	***r* = −0.52, *p* < 0.01**

Significant correlations in bold.

**Figure A1:**
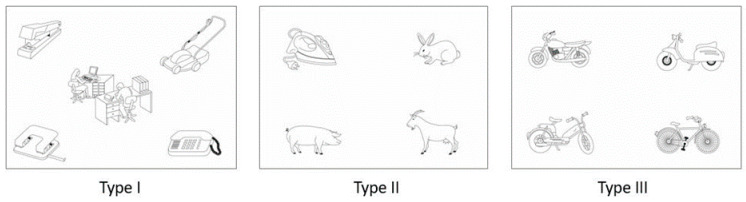
NVST subtest—Semantic Sorting: Examples of the three different task types.

**Figure A2:**
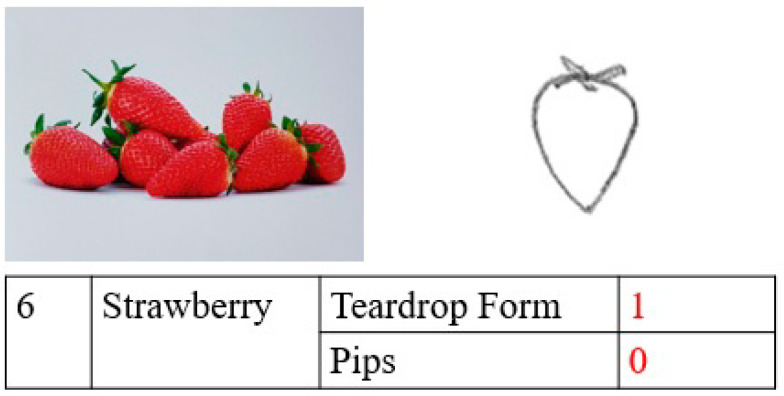
Drawing Task: Model-photo (**left**) and a drawing by a PWA (**right**). The strawberry-item comprises two obligatory features, i.e., the teardrop form and the presence of pips (**bottom**). The example is awarded a score of 1, because the pips are missing.

**Figure A3:**
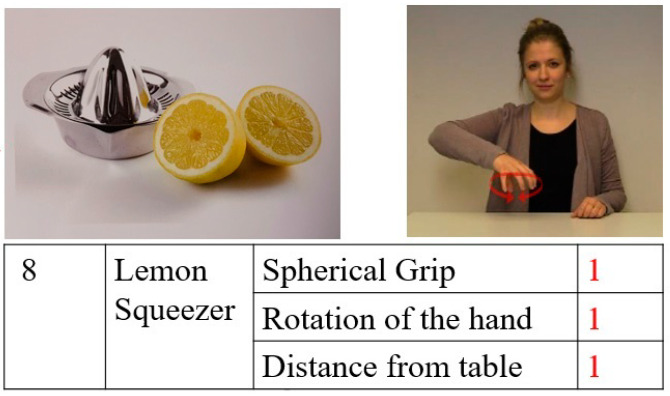
Example Scoring Pantomime task

## Data Availability

The data presented in this study are available on request from the corresponding author. The data are not publicly available due to privacy reasons.

## Appendix A

The Nonverbal Semantics Test (NVST)

Objectives: The Nonverbal Semantics Test [43] is a standardized tool for the clinical assessment of semantic processing disorders in persons with neurological disorders (CVA and neurodegenerative disease). The major objective of the test is to capture semantic processing capacities using tasks that do not draw on verbal capacities—Semantic Sorting, Drawing, and Pantomime to command. As the application is independent of spoken or written linguistic processing capacities, the NVST is also suitable for participants with severe aphasia. Results allow comparisons between the subtests and indicate if particular nonverbal resources are intact and can be used for successful functional communication. The results may form the basis for a tailored therapy planning and can be used for monitoring therapy outcomes. In persons with dementia, the test may support the differential diagnostics of dementia subtypes.

The test allows for the detection of the presence of semantic impairment and the determination of level of severity. In its current form it has so far been applied to persons with aphasia after CVA and to persons with neurodegenerative disorders (dementia-type Alzheimer’s and frontotemporal dementia). An application to other patient groups is conceivable.

Design: To develop the test, three clinically established tasks [10,17] were revised and integrated into a comprehensive assessment tool. The first subtest involves a Semantic Sorting task based on an odd-one-out paradigm, consisting of revisions of three of the five subtests of the former Bogenhausener Semantik-Untersuchung [10]. Tasks are self-explanatory and require the participants to make decisions about semantic relationships between pictured objects and/or situations. Participants give their answer by pointing. Raw scores are derived from error counts of the 24 items.

Examples for the items of the three different task types (each *n* = 8) are illustrated in Figure A1. Task type I (left) requires a matching of objects to a situational context in which they may occur. As an illustration, in the left panel of Figure A1 four objects are arranged around an office scenario in the middle. The participant has to point at the object that does NOT fit this situation (lawnmower vs. telephone, hole punch, and stapler). In particular, the PWA should benefit from a given situational context in task type I [82].

In the second (II) and the third (III) task, each item consists of four pictures. Again, the participant has to show the object that does NOT fit (e.g., type II: iron vs. pig, rabbit, and goat). Task types II and III differ from each other with respect to the semantic proximity of the target and the foils. Semantic relationships in task III are closer. Hence, the conception of the tasks represents an increasing degree of difficulty, which had been confirmed using the original Bogenhausener Semantik-Untersuchung in a group of 71 PWA [10] (p. 15).

The NVST subtest Drawing is a revised version of the drawing-from-memory task of the Pantomime and Drawing Test [17]. Participants are asked to draw 12 objects. During the instruction, a photo of the respective object is briefly shown to the participant to ensure understanding. To prevent direct copying, the pictured objects are shown in unusual perspectives or several representatives of the objects are displayed. After removal of the photo, the participant is asked to draw the object. Raw scores are awarded according to the representation of pre-defined features in the participant’s drawings. The quality of the drawings is not taken into account for scoring. A total of thirty features are defined. Figure A2 illustrates the scoring for the object strawberry.

The subtest Pantomime of the NVST is a revised version of the Pantomime task of the Pantomime- and Drawing Test [17]. Participants are asked to pantomime the use of 12 objects. During the instruction, a picture of the respective object is briefly shown to the participant to ensure understanding. The scoring is conducted using a total of 30 pre-defined features. An example for the scoring of the item Lemon squeezer is given in Figure A3.

Psychometrics: Psychometric properties were determined using NVST data [43] from a sample of 82 persons with neurologic conditions, i.e., 34 with aphasia after stroke and 48 with dementia (29–87 years, 49 women).

Calculation of norms: Differential norms were developed based on data from 192 neurologically healthy participants stratified for age (20–39, 40–59, and 60–85 years), gender, and education (with/without high school graduation). Generalized linear mixed models (logit) were calculated for each subtest to determine the stratification factors that had a significant influence on the healthy participants’ performance. No stratification was required for the Semantic Sorting subtest, but a stratification by education for the Drawing subtest, and a stratification by age and gender for the Pantomime subtest.

For each subtest and each stratification subgroup, a table is provided which lists the threshold of impaired test performance (5th percentile) and a coarse metric based on the distance between the median value (corresponding to a normalized score of 0) and the 5th percentile (corresponding to a normalized score of 1). Thus, normalized test scores > 1 indicate impaired performance, with higher scores indicating greater impairment. Similar to a z-score normalization, the units of this non-parametric norm depend on a central tendency measure as origin and a dispersion measure as distance unit of each raw parameter’s distribution in the respective stratification group of the calibration sample. The transformation makes the scores comparable across age, gender, and education groups as well as across the three subtests.

Furthermore, coarse severity levels can be distinguished (Table A1). These levels were not derived empirically but turned out in retrospect to yield similar severity classifications of an NVST evaluation sample of 82 patients with neurologic conditions across the three subtests.

**Table A1:** Severity levels.

Normalized Scores	Interpretation
<1	No impairment
≥1 and <2	Mild impairment
≥2 and <4	Moderate impairment
≥4	Severe impairment

Interrater reliability: Data from 30 participants (10 neurologically healthy, 10 persons with aphasia after stroke, 10 persons with dementia, quasi-randomly selected) was analysed independently by two trained raters. Agreement was determined at item level (*n* = 900). A reliability analysis using Cohen’s Kappa revealed agreement scores of κ = 0.842 (95% CI: 0.765, 0.967) for Drawing and κ = 0.798 (95% CI: 0.905–0.979) for Pantomime, indicating almost perfect or substantial agreement, respectively, according to the criteria of Landis and Koch [83]. Since the scoring of the Semantic Sorting subtest is limited to pointing error counts, a calculation of interrater reliability was immaterial.

Internal consistency: Cronbach’s alpha, calculated across the patient group (*n* = 82) was 0.83 (95% CI: 0.75–0.89) for Semantic Sorting, 0.91 (95% CI: 0.87–0.94) for Drawing, and 0.92 (95% CI: 0.88–0.95) for Pantomime, indicating reasonably good consistency for all NVST-subtests.

Retest reliability: A two-way mixed effects model (absolute agreement) revealed reasonable single measure intraclass correlations (ICC) for a subgroup of persons with neurological conditions (*n* = 12; Semantic Sorting: ICC = 0.887 with 95% confidence interval 0.668–0.966; Drawing: ICC = 0.873 with 95% confidence interval 0.612–0.964; Pantomime: ICC = 0.960 with 95% confidence interval 0.868–0.988). There were no systematic differences between the scores of the two assessment points (Wilcoxon signed rank test, all |Z| ≤ 1.260, *p* ≥ 0.208).

Validity: All three subtests conformed to standard criteria of content validity and criterion validity. In a principal component analysis, the items of the three subtests clearly clustered into three almost equally weighted factors corresponding with the three NVST subtests, demonstrating that Semantic Sorting, Drawing, and Pantomime actually probe different constructs. Furthermore, a comparison with the Pyramids and Palm Trees Test, PPT [7] through a multiple linear regression analysis revealed that only the subtest Semantic Sorting had a significant influence on the PPT scores of 33 patients with neurologic conditions (beta = −2.57, *p* < 0.01). This is a reasonable result, because the PPT and the Semantic Sorting subtest of the NVST are both picture-based tasks that require the identification of semantic relationships.

One aspect of discriminant validity that should be mentioned in more detail relates to the prerequisite that test performance—especially for the subtests Drawing and Pantomime—should be independent of manual skills. This is particularly important for test applications in persons with aphasia after left hemisphere damage, most of whom are dependent on their non-dominant left hand due to a right upper limb paresis. To investigate the influence of the use of the dominant or non-dominant hand, respectively, eighty-seven of the 192 neurologically healthy participants from the calibration sample (45%) had been asked to perform the task with their non-dominant hand. Separate generalized linear mixed models (GLMM, logit link) were calculated for Drawing and Pantomime, with items and participants as random effects (intercept) and sex, education, age, and hand dominance as fixed effects. In neither model did the factor hand dominance have a significant effect (|β| < 0.25). Furthermore, two comparative GLMMs in which the hand dominance factor was not modelled were equivalent with the two full models (χ^2^ < 3.5 in both Drawing and Pantomime), which demonstrates that the use of the non-preferred hand does not provide a disadvantage in the two NVST subtests that rely on hand motor functions.

A detailed description of the psychometric properties of the NVST subtests is documented in the test manual [43].

## References

[1] Morris J.C., Heyman A., Mohs R.C., Hughes J.P., van Belle G., Fillenbaum G., Mellits E.D., Clark C. (1989). The Consortium to Establish a Registry for Alzheimer’s Disease (CERAD). Part I Clinical and neuropsychological assessment of Alzheimer’s disease. Neurology.

[2] Nasreddine Z.S., Phillips N.A., Bédirian V., Charbonneau S., Whitehead V., Collin I., Cummings J.L., Chertkow H. (2005). The Montreal Cognitive Assessment, MoCA: A brief screening tool for mild cognitive impairment. J. Am. Geriatr. Soc..

[3] Huber W., Poeck K., Weniger D., Willmes K. (1983). Aachener Aphasie Test.

[4] Kertesz A. (2007). The Western Aphasia Battery—Revised.

[5] Blumstein S., Baker E., Goodglass H. (1977). Phonological factors in auditory comprehension in aphasia. Neuropsychologia.

[6] Robson H.R., Griffiths T.D., Grube M., Woollams A.M. (2019). Auditory, phonological, and semantic factors in the recovery from Wernicke’s Aphasia poststroke: Predictive value and implications for rehabilitation. Neurorehabilit. Neural Repair.

[7] Howard D., Patterson K. (1992). Pyramids and Palmtree Test.

[8] Bozeat S., Lambon Ralph M.A., Patterson K., Garrard P., Hodges J.R. (2000). Non-verbal semantic impairment in semantic dementia. Neuropsychologia.

[9] Adlam A.-L.R., Patterson K., Bozeat S., Hodges J.R. (2010). The Cambridge Semantic Memory Test Battery: Detection of semantic deficits in semantic dementia and Alzheimer’s disease. Neurocase.

[10] Glindemann R., Klintwort D., Ziegler W., Goldenberg G. (2002). Bogenhausener Semantik—Untersuchung (BOSU): Manual.

[11] Gainotti G. (2020). Drawing and gesturing in aphasia. Neuropsychol. Trends.

[12] Trojano L., Gainotti G. (2016). Drawing disorders in alzheimer’s Disease and other forms of dementia. J. Alzheimer’s Dis..

[13] Bozeat S., Lambon Ralph M.A., Graham K.S., Patterson K., Wilkin H., Rowland J., Rogers T.T., Hodges J.R. (2003). A duck with four legs: Investigating the structure of conceptual knowledge using picture drawing in semantic dementia. Cogn. Neuropsychol..

[14] Brantjes M., Bouma A. (1991). Qualititative Analysis of the Drawings of Alzheimer’s Patients. Clin. Neuropsychol..

[15] Pozueta A., Lage C., Martinez M.G., Kazimierczak M., Bravo M., Lopez-Garcia S., Riancho J., Gonzalez-Suarez A., Vazquez-Higuera J.L., de Arcocha-Torres M. (2019). A Brief Drawing Task for the Differential Diagnosis of Semantic Dementia. J. Alzheimer’s Dis..

[16] Gainotti G., Silveri M.C., Villa G., Caltagirone C. (1983). Drawing objects from memory in aphasia. Brain.

[17] Goldenberg G., Hartmann K., Schlott I. (2003). Defective pantomime of object use in left brain damage: Apraxia or asymbolia?. Neuropsychologia.

[18] Vanbellingen T., Kersten B., Van Hemelrijk B., Van de Winckel A., Bertschi M., Müri R., De Weerdt W., Bohlhalter S. (2010). Comprehensive assessment of gesture production: A new test of upper limb apraxia (TULIA). Eur. J. Neurol..

[19] Randerath J., Buchmann I., Liepert J., Büsching I. (2017). Diagnostic Instrument for Limb Apraxia (DILA-S): Manual.

[20] Goldenberg G., Hermsdörfer J., Glindemann R., Rorden C., Karnath H.O. (2007). Pantomime of Tool Use Depends on Integrity of Left Inferior Frontal Cortex. Cereb. Cortex.

[21] Goldenberg G. (2017). Facets of pantomime. J. Int. Neuropsychol. Soc..

[22] Van Nispen K., van de Sandt-Koenderman M., Mol L., Krahmer E. (2016). Pantomime production by people with aphasia: What are the influencing factors?. J. Speech Lang. Hear. Res..

[23] Goldenberg G. (2013). Apraxia—The Cognitive Side of Motor Control.

[24] Davis G.A., Wilcox M.J., Chapey R. (1981). Icorporating Parameters of Natural Conversation in Aphasia Treatment. Language Intervention Strategies in Adult Aphasia.

[25] Nobis-Bosch R., Bruehl S., Krzok F., Jakob H., van de Sandt-Koenderman M., van der Meulen I. (2020). Szenario-Test. Testung Verbaler und Non-Verbaler Aspekte Aphasischer Kommunikation.

[26] Van der Meulen I., Van de Sandt-Koenderman W.M.E., Duivenvoorden H.J., Ribbers G.M. (2010). Measuring verbal and non-verbal communication in aphasia: Reliability, validity, and sensitivity to change of the Scenario Test. Int. J. Lang Commun. Disord..

[27] Van der Meulen I., van Gelder-Houthuizen J., Wiegers J., Wielaert S., van de Sandt-Koenderman M. (2008). Scenario Test: Verbale en non-Verbale Communicatie bij Afasie. Handleiding Scenario-Test.

[28] Glindemann R., Zeller C., Ziegler W. (2018). Kommunikativ-Pragmatisches Screening (KOPS).

[29] Raymer A.M., Singletary F., Rodriguez A., Ciampitti M., Heilman K.M., Rothi L.J. (2006). Effects of gesture+verbal treatment for noun and verb retrieval in aphasia. J. Int. Neuropsychol. Soc..

[30] Kinney J., Wallace S.E., Schreiber J.B. (2020). The relationship between word retrieval, drawing, and semantics in people with aphasia. Aphasiology.

[31] Hung P.-F., Ostergren J. (2019). A comparison of drawing and writing on facilitating word retrieval in individuals with aphasia. Aphasiology.

[32] Ferguson N.F., Evans K., Raymer A.M. (2012). A comparison of intention and pantomime gesture treatment for noun retrieval in people with aphasia. Am. J. Speech Lang. Pathol..

[33] Marangolo P., Bonifazi S., Tomaiuolo F., Craighero L., Coccia M., Altoè G., Provinciali L., Cantagallo A. (2010). Improving language without words: First evidence from aphasia. Neuropsychologia.

[34] Murteira A., Nickels L. (2020). Can gesture observation help people with aphasia name actions?. Cortex.

[35] Rose M.L., Attard M.C., Mok Z., Lanyon L.E., Foster A.M. (2013). Multi-modality aphasia therapy is as efficacious as a constraint-induced aphasia therapy for chronic aphasia: A phase 1 study. Aphasiology.

[36] Wallace S.E., Purdy M., Skidmore E. (2014). A multimodal communication programm for aphasia during inpatient rehabilitation: A case study. NeuroRehabilitation.

[37] Rose M.L., Copland D., Nickels L., Togher L., Meinzer M., Rai T., Cadilhac D.A., Kim J., Foster A., Carragher M. (2019). Constraint-induced or multi-modal personalized aphasia rehabilitation (COMPARE): A randomized controlled trial for stroke-related chronic aphasia. Int. J. Stroke.

[38] Roper A., Marshall J., Wilson S. (2016). Benefits and Limitations of Computer Gesture Therapy for the Rehabilitation of Severe Aphasia. Front. Hum. Neurosci..

[39] Lambon Ralph M.A., Jefferies E., Patterson K., Rogers T.T. (2017). The neural and computational bases of semantic cognition. Nat. Rev. Neurosci..

[40] Rogers T.T., Lambon Ralph M.A., Garrard P., Bozeat S., McClelland J.L., Hodges J.R., Patterson K. (2004). Structure and deterioration of semantic memory: A neuropsychological and computational investigation. Psychol. Rev..

[41] Patterson K., Lambon Ralph M.A., Hickok G., Small S.L. (2016). The hub-and-spoke hypothesis of semantic memory. Neurobiology of Language.

[42] Patterson K., Nestor P.J., Rogers T.T. (2007). Where do you know what you know? The representation of semantic knowledge in the human brain. Nat. Rev. Neurosci..

[43] Hogrefe K., Glindemann R., Ziegler W., Goldenberg G. Nonverbaler Semantiktest (NVST).

[44] Miller N., Willmes K., De Bleser R. (2000). The psychometric properties of the English language version of the Aachen Aphasia Test (EAAT). Aphasiology.

[45] The R Development Core Team (2020). A Language and Environment for Statistical Computing.

[46] Fox J., Weisberg S. (2019). An {R} Companion to Applied Regression.

[47] James G., Witten D., Hastie T., Tibshirani R. (2013). An Introduction to Statistical Learning: With Applications in R.

[48] Revelle W.R. (2020). Psych: Procedures for Personality and Psychological Research.

[49] Finkel L., Hogrefe K., Frey S.H., Goldenberg G., Randerath J. (2018). It takes two to pantomime: Communication meets motor cognition. Neuroimage Clin..

[50] Gentilucci M., Corballis M.C. (2006). From manual gesture to speech: A gradual transition. Neurosci. Biobehav. Rev..

[51] Arbib M.A. (2012). How the Brain Got Language: The Mirror System Hypothesis.

[52] Tomasello M. (2008). Origins of Human Communication.

[53] Mirman D., Chen Q., Zhang Y., Wang Z., Faseyitan O.K., Coslett H.B., Schwartz M.F. (2015). Neural organization of spoken language revealed by lesion-symptom mapping. Nat. Commun..

[54] Vandenberghe R., Price C.J., Wise R., Josephs O., Frackowiak R.S.J. (1996). Functional anatomy of a common semantic system for words and pictures. Nature.

[55] Mummery C.J., Patterson K., Hodges J.R., Price C.J. (1998). Functional neuroanatomy of the semantic system: Divisible by what?. J. Cogn. Neurosci..

[56] Binder J.R., Desai R.H. (2011). The neurobiology of semantic memory. Trends Cogn. Sci..

[57] Gainotti G. (2015). Is the difference between right and left ATLs due to the distinction between general and social cognition or between verbal and non-verbal representations?. Neurosci. Biobehav. Rev..

[58] Butler C.R., Brambati S.M., Miller B.L., Gorno-Tempini M.L. (2009). The neural correlates of verbal and non-verbal semantic processing deficits in neudegenerative disease. Cogn. Behav. Neurol..

[59] Snowden J.S., Thompson J.C., Neary D. (2012). Famous people knowledge and the right and left temporal lobes. Behav. Neurol..

[60] Woollams A.M., Patterson K. (2018). Cognitive consequences of the left-right assymetry of atrophy in semantic dementia. Cortex.

[61] Harrington G.S., Farias D., Davis C. (2009). The neural basis for simulated drawing and the semantic implications. Cortex.

[62] Farias D., Davis C., Harrington G. (2006). Drawing: Its contribution to naming in aphasia. Brain Lang..

[63] Makuuchi M., Kaminaga T., Sugishita M. (2003). Both parietal lobes are involved in drawing: A functional MRI study and implications for constructional apraxia. Cogn. Brain Res..

[64] Harrington G.S., Farias D., Davis C.H., Buonocore M.H. (2007). Comparison of the neural basis for imagined writing and drawing. Hum. Brain Mapp..

[65] Grossmann M. (1988). Drawing deficits in brain-damaged patients’ freehand pictures. Brain Cogn..

[66] Lausberg H., Zaidel E., Cruz R.F., Ptito A. (2007). Speech-independent production of communicative gestures: Evidence from patients with complete callosal disconnection. Neuropsychologia.

[67] Hogrefe K., Rein R., Skomroch H., Lausberg H. (2016). Co-speech hand movement behaviour in narrations: What is the impact of right vs. left hemisphere damage?. Neuropsychologia.

[68] Blonder L.X., Burns A.F., Bowers D., Moore R.W., Heilman K.M. (1995). Spontaneous gestures following right hemisphere infarct. Neuropsychologia.

[69] Cocks N., Hird K., Kirsner K. (2007). The relationship between right hemisphere damage and gesture in spontaneous discourse. Aphasiology.

[70] Akbiyik S., Karaduman A., Göksun T., Chatterjee A. (2018). The relationship between co-speech gesture production and macrolinguistic disourse abilities in people with focal brain injury. Neuropsychologia.

[71] Buxbaum L.J., Shapiro A., Coslett H.B. (2014). Critical brain regions for tool related and imitative actions: A componential analysis. Brain.

[72] Goldenberg G., Randerath J. (2015). Shared neural substrates of aphasia and apraxia. Neuropsychologia.

[73] Hogrefe K., Ziegler W., Glindemann R., Klonowski M., Wagner-Sonntag E., Klingenberg G., Diehl-Schmid J., Roßmeier C., Danek A., Levin J. (2019). Application of the Nonverbal Semantics Test (NVST) to persons with aphasia after stroke and persons with dementia. Stem Spraak Taalpathol..

[74] Hogrefe K., Ziegler W., Weidinger N., Goldenberg G. (2012). Non-verbal communication in severe aphasia: Influence of aphasia, apraxia, or semantic processing?. Cortex.

[75] Hogrefe K., Ziegler W., Weidinger N., Goldenberg G. (2017). Comprehensibility and neural substrate of communicative gestures in severe aphasie. Brain Lang..

[76] Hogrefe K., Ziegler W., Wiesmayer S., Weidinger N., Goldenberg G. (2013). The actual and potential use of gestures for communication in aphasia. Aphasiology.

[77] Caute A., Pring T., Cocks N., Cruice M., Best W., Marshall J. (2013). Enhancing communication through gesture and naming therapy. J. Speech Lang. Hear. Res..

[78] Bauer A., Kaiser G. (1995). Drawing on drawings. Aphasiology.

[79] Warrington E.K., Cipolotti L. (1996). Word comprehension. The distinction between refractory and storage impairments. Brain.

[80] Jefferies E., Lambon Ralph M.A. (2006). Semantic impairment in stroke aphasia versus semantic dementia: A case-series comparison. Brain.

[81] Chapman C.A., Hasan O., Schulz P.E., Martin R.C. (2020). Evaluating the distinction between semantic knowledge and semantic access: Evidence from semantic dementia and comprehension-impaired stroke aphasia. Psychon. Bull. Rev..

[82] Kelter S., Cohen R., Engel D., List G., Strohner H. (1976). Aphasic disorders in matching tasks involving conceptual analysis and covert naming. Cortex.

[83] Landis J.R., Koch G.G. (1977). The measurement of observer agreement for categorical data. Biometrics.

